# TiO_2_–SrTiO_3_ Biphase Nanoceramics as Advanced Thermoelectric Materials

**DOI:** 10.3390/ma12182895

**Published:** 2019-09-07

**Authors:** Alexey Zavjalov, Sergey Tikhonov, Denis Kosyanov

**Affiliations:** School of Natural Sciences, Far Eastern Federal University, 8 Sukhanova Street, Vladivostok 690950, Russian Federation

**Keywords:** SrTiO_3_, TiO_2_, biphase nanoceramics, two-dimensional electron gas, 2DEG, thermoelectric efficiency, thermoelectric efficiency *ZT*, spark plasma sintering, SPS, oxide thermoelectric materials

## Abstract

The review embraces a number of research papers concerning the fabrication of oxide thermoelectric systems, with TiO_2_−SrTiO_3_ biphase ceramics being emphasized. The ceramics is particularly known for a two-dimensional electron gas (2DEG) forming spontaneously on the TiO_2_/SrTiO_3_ heterointerface (modulation doping), unlike ordinary 2DEG occurrence on specially fabricated thin film. Such effect is provided by the SrTiO_3_ conduction band edge being 0.40 and 0.20 eV higher than that for anatase and rutile TiO_2_, respectively. That is why, in the case of a checkered arrangement of TiO_2_ and SrTiO_3_ grains, the united 2D net is probably formed along the grain boundaries with 2DEG occurring there. To reach such conditions, there should be applied novelties in the field of ceramics materials science, because it is important to obtain highly dense material preserving small (nanoscale) grain size and thin interface boundary. The review also discusses some aspects of reactive spark plasma sintering as a promising method of preparing perovskite-oxide TiO_2_−SrTiO_3_ thermoelectric materials for high-temperature applications.

## 1. Introduction

Today, a large part of energy is produced by heat machines leading to a high amount of heat being dissipated (60% of total energy) into the environment due to known physical limitations [1]. Therefore, secondary exhaust heat processing is highly promising for energy efficiency improvement. One of the ways to do that is the direct consumption of exhaust heat, which is the valuable resource itself, right in the power plants. However, this method is rather limited [2] in its ability to satisfy growing energy consumption and to reduce the usage of conventional fuels, thus, new alternative and effective ways to convert the excess heat into energy are in high demand.

**Figure 1:**
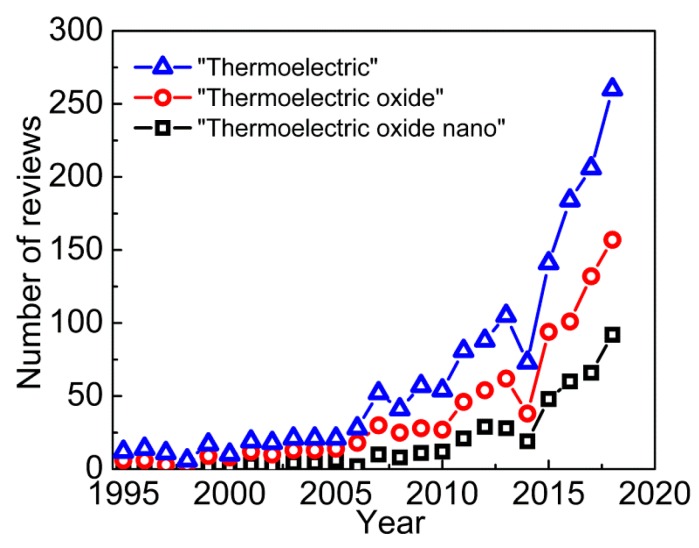
Statistics on review papers from 1995 to 2018 on ScienceDirect on queries “Thermoelectric”, “Thermoelectric oxide” and “Thermoelectric oxide nano”.

Special attention in this respect is given to technologies of direct heat conversion into electric power [3,4,5,6]. Experimental and theoretical investigations in order to understand the mechanisms involved and to improve the materials properties and conversion efficiency have been ongoing for more than half a century. Particularly, Figure 1 shows the statistics of review papers from 1995 to 2018 on “Thermoelectric”, “Thermoelectric oxide” and “Thermoelectric oxide nano” queries. The annual amount of research has increased more than 20-fold (from 12 in 1995 to 260 in 2018). The highest publication activity in this field is happening now: From 2014, the number of published reviews grew by 40 every year, which corresponds to additional 1000 research papers. They are focused mainly on the thermoelectric properties of oxides and thermoelectric devices based on them (over 50% of papers have studied oxide systems since 2014) (Figure 1).

Historically, thermoelectric energy conversion started in 1821, when T.J. Seebeck described the occurrence of voltage on the ends of conductor that are exposed to different temperatures [7]. To some extent, the reverse effect of occurring temperature difference was observed by J.C.A. Peltier for the heterometallic circuit under voltage [8]. Both effects are named after their discoverers and serve as a basis for modern devices of secondary processing of exhaust heat and for heat pumps.

The coefficient of power efficiency for the thermoelectric converter *η* is evaluated as follows Equation (1) [9,10,11]:

(1) $$\eta =\frac{T_{h}-T_{c}}{T_{h}}\frac{M-1}{M+\left(T_{c}/T_{h}\right)},$$

(2) $$M=\sqrt{1+Z\left(T_{h}+T_{c}\right)/2},$$

where *T_h_* and *T_c_*—temperature of hot and cold side, respectively; *Z*—thermoelectric quality factor.

At the same time, the key thermoelectric feature is the thermoelectric efficiency *ZT*, which is a dimensionless value [12]. The most widely used thermoelectrics at the moment, Bi_2_Te_3_ and PbTe, are characterized by *ZT*~1 that determines the borderline of the application of these materials on a large scale [13,14]. Additionally, alloys based on heavy metals with *ZT* > 1 are obtained, thus, expanding the horizons of thermoelectric generators applications [14,15,16,17,18,19,20,21,22,23]. However, many technological processes produce exhaust heat at high temperatures (the temperature of an exhaust pipe is ~700 °C). Under such conditions, partial thermal decomposition of the mentioned materials takes place leading to contamination of the environment with wastes containing heavy and/or toxic metals. Radioisotope thermoelectric generators (RITEGs) are a good example of the solution to this problem, where heat of radioactive decomposition is converted into electric energy. The striking example of using RITEG is space probe “Voyager-2”, the most distant evidence of human beings out of Earth. There is SiGe-based semiconductor thermoelectric material installed on “Voyager-2”, which produces electricity from the heat of the plutonium core at 1000 °C with the *ZT* value reaching 1 only at such elevated temperatures [24]. Similar systems of autonomous power supply are used in many other ground-level frames (radio beacon, weather stations and etc.). It is noteworthy that indicated temperatures are extreme for silicide materials [5], i.e., there is a need for thermoelectric materials that are stable at such temperatures (and radiation background).

Above 1000 °C, oxide compounds are well thermally and chemically stable. They can be applied either as separate materials [10,11,25] or as a high-temperature layer in the composite thermoelectric systems [11]. J.R. Szczech et al. have shown that thermoelectric efficiency can be drastically enhanced if nanostructured materials are used [26]. The last decade’s achievements and prospects are fully reported in Chapters 22, 23 of the review “Nanotechnology for Energy Sustainability” [5]. Special attention should be paid to the work by H. Ohta et al., which presents a pulse laser sputtering method for SrTiO_3_/TiO_2_ bi-layered system fabrication [27]. The main feature of that composite is that two-dimensional electron gas (2DEG) occurs not in the specially formed thin layer as usual, but in the interface region of SrTiO_3_/TiO_2_ ceramics. Therefore, if grains of SrTiO_3_ and TiO_2_ are checkered, then the unified coherent 2D surface is obtained along the grain boundaries providing 2DEG formation. But there is no research addressing this issue. A similar structure is proposed by K. Koumoto et al. only as a hypothesis [10]. The authors recommend a preparation of SrTiO_3_ material with 10 at.% of La ions in Sr positions being separated by thin layers of 20 at.% of Nb ions in Ti positions. Alternatively, similar material should be obtained as a ceramic that utilizes 2DEG features in thin layers. In that case, ceramic novelties should be applied to achieve minimal thickness of the separation layer. Anyway, the adaptation of methods of large-scale production for ceramics can provide available and highly efficient thermoelectric materials for a broad range of practical applications. In view of the above, the review is devoted to the search of a new approach to fabricate thermoelectric oxide materials based on bi-phase SrTiO_3_-based ceramics. This review will ultimately attempt to answer the question, "Is it possible to further improve the thermoelectric properties of SrTiO_3_-based ceramics?"

## 2. Oxide Thermoelectrics History

H. Ohta distinguished the three main periods of oxide thermoelectric research [1]. The first papers published in the 1950s–1970s studied thermoelectric characteristics of simple oxides, such as CdO [28], NiO [29], ZnO [30], In_2_O_3_ [31], SrTiO_3_ [32], rutile-TiO_2_ [33], SnO_2_ [34], and Cu_2_O [35]. In 1986, two IBM employees, K. Muller and G. Bednorz, discovered high-temperature superconductivity for the La_2−x_Ba_x_CuO_4_ system [36] and consequently won the Nobel Prize in physics in 1987. However, the real breakthrough in a plethora of fields occurred after the discovery of superconductivity in the YBa_2_Cu_3_O_7−x_ system (77 K) in 1987, because fairly cheap liquid nitrogen enabled researchers to achieve a superconductive state in that case [37]. Then, the second stage of studying thermoelectric properties of high-temperature superconductive oxides began: La_2_CuO_4_ [38], La–Ba–Cu–O [39], YBa_2_Cu_3_O_7−δ_ [40], Tl–Ca–Ba–Cu–O [41] and etc. The third stage in thermoelectric oxide research demonstrated high *ZT* values for simpler oxide systems as CaMnO_3_ [42], Al-doped ZnO [43], Na_x_CoO_2_ [44], Ca_3_Co_4_O_9_ (Ca_2_Co_2_O_5_) [45,46] and electron-doped SrTiO_3_ [47,48,49,50]. Data on these systems presented up to 2012 show their *ZT* values were still below 1 [50]: Ca_3_Co_4_O_9_ (0.15–0.5 at 1000 K), Na_x_CoO_2_ (0.3–0.9 at 950 K), SrTiO_3_ (0.2–0.35 at 1000 K), CaMnO_3_ (0.1–0.2 at 1000 K), and ZnO (0.03–0.5 at 1073 K).

The general trend in research has been shifting towards low-sized (including nano-) thermoelectric systems for more than 20 years already (Figure 1). Apparently, that has happened because the “classical” methods of improving oxide’s thermoelectric characteristics (e.g., doping with rare earth metals and/or creating point defects) have become nearly exhausted. At the same time, new effects have been observed on a nanoscale, namely, two-dimensional electron gas (2DEG) can be formed in the material. In other words, we are observing the fourth stage of oxide thermoelectric research at the moment.

## 3. Theoretical Aspects of Thermoelectricity

As it was mentioned above Equations (1) and (2), thermoelectric efficiency of the material is often assessed in terms of figure of merit *ZT* (Equation (3)) [12]:

(3) $$ZT=S^{2}\sigma T/\kappa ,$$

where *S* is Seebeck’s coefficient (thermal emf); *σ* is electrical conductivity; *κ* is thermal conductivity.

Improving the *ZT* value (Equation (3)) boils down to increasing the power factor (*S*^2^*σ*) and reducing thermal conductivity *κ*. Although theoretical limit for *ZT* is absent [51], thermoelectric parameters in practice are deeply connected with each other and improvement in one worsens the other, thus, having no effect on the *ZT* value.

The main contribution to thermal conductivity comes from phonons and charge carriers, that is why the whole thermal conductivity of the materials is a sum of their partial contributions (Equation (4)) [52]:

(4) $$\kappa =\kappa_{ph}+\kappa_{e},$$

Gas kinetic theory for solids determines electron and phonon thermal conductivity according to general principles (Equations (5) and (6)) [52]:

(5) $$\kappa_{ph}=\frac{1}{3}C_{v}v_{s}L_{ph},$$

(6) $$\kappa_{e}=\frac{1}{3}c_{v}v\Lambda ,$$

where *v_s_*—sound velocity; *C_v_*—thermal capacity at constant volume; *L_ph_*—phonon’s mean free path (MFP) controlled by phonon-phonon distance [53]; *c_v_*—electrons’ specific thermal capacity per unit volume; *v*—mean electron velocity that can be taken as Fermi velocity *v_F_*; Λ—electron’s mean free path.

According to the Wiedemann–Franz relationship [54], electron thermal conductivity (Equation (6)) can be given by:

(7) $$\kappa_{e}=L\sigma T,$$

where *L*—Lorentz number 2.45 × 10^−8^ V^2^/K^2^.

As it follows from Equation (7), electrical conductivity decreases with electron thermal conductivity. However, acoustic phonons *κ_ph_* contributes more to thermal conductivity (Equation (4)) in case of semiconductors and insulators (although its value can be lower as compared to electron conductivity in highly alloyed and non-stoichiometric systems) [52].

Phonon thermal conductivity is the only parameter which is not defined by electron structure and, therefore, does not depend on other parameters [55]. That is why many researchers try to minimize it close to theoretical value (0.25–0.50 Wm^−1^∙K^−1^) via confining phonon MFP down to interatomic distance [56]. It is noteworthy that idea of improving the *ZT* value via simultaneous increasing electrical conductivity *σ* and decreasing phonon thermal conductivity *κ_ph_* serves as a basis for the “Phonon glass–electron crystal” (PGEC) concept [3]. PGEC materials work as crystals for electrons, providing good conductivity, and dissipate phonons as well, like glasses, thus reducing phonon thermal conductivity. The theoretical *ZT* value of ~4 at 77–300 K for such materials is given in [51]. However, the *ZT* value for such systems does exceed 1 due to the introduction of special sites dissipating phonons (such as skutterudites, clathrates, and zintle phases) into the material’s bulk [5].

Obviously, there is no formula connecting the phonon thermal conductivity *κ_ph_* with other thermoelectric characteristics in the presented review. In practice, thermoelectric characteristics are often affected by the material’s structure, which has a complex effect on all the parameters of the material as a thermoelectric. From this point of view, let us consider several experimental works studying SrTiO_3_-based materials that are an objective of the present review.

Reference [52] (referring to [10]) indicates that low ZT values for SrTiO_3_-based materials are caused by their high thermal conductivity *κ_ph_*. This work also notes that point defects of alloying agents are not effective phonon dissipation sites [52]. Therefore, changing the power factor (*S*^2^*σ*) by introducing ligands can be done without an impact on thermal conductivity *κ_ph_*. As an efficient way to reduce thermal conductivity *κ*, H. Muta et al. suggested to provide structural defects via substitution of Sr^2+^ with Ca^2+^ or Ba^2+^ in titanates solid solutions, because this type of defect will act as phonon dissipation sites [57,58]. However, such substitution may lead to significant asymmetry with thermal conductivity reduction being compensated by lowering the power factor *S*^2^*σ* [59]. At the same time, semiisovalent substitution of Sr^2+^ with Eu^2+^ has practically no effect on power factor *S*^2^*σ* and reduces phonon’s MFP by ~12%, but considerable decrease of the overall thermal conductivity is observed only at *T* < 400K [60]. Such type of substitution is unusable because oxide compounds are considered as promising thermoelectric materials for high-temperature applications (>1000 °C).

Special attention should be paid for double alloying of SrTiO_3_-based thermoelectrics. Diversity of dissipation sites in the crystal lattice always enhances thermal conductivity reduction *κ*: (Sr_1−x−y_La_x_Yb_y_)TiO_3_ [61], Sr_1−x_(Ti_1−y−z_Nb_y_W_z_)O_3±δ_ [62], (Sr_1−x_La_x_)(Ti_1−x_Ta_x_)O_3_ and (Sr_1−x_La_x_)(Ti_1−x_Nb_x_)O_3_ [63]. On the other hand, conductivity and Seebeck’s coefficient are also affected in a way depending on the quantity and/or the type of point defects. More in-depth information on alloying effects on thermoelectric properties can be found in some review papers [11,52,64].

Additionally, phonon dissipation to suppress *κ_ph_* is not an ultimate goal. Modelling has shown [65] that there are spectrally different groups of phonons responsible for heat transfer and electron drag towards the temperature gradient. The latter phonons contribute more or less significantly to thermo-emf (Seebeck effect) *S* at least at low temperatures. The phonon drag contribution to thermo-emf *S* is often observed for electron-doped systems [27,32,47,66]. Therefore, when developing thermoelectric materials, it is important to prepare filters suppressing heat transferring phonons and transmitting electron dragging ones that can enhance the *ZT* value of the material (at least for low temperatures) [65].

Power factor (*S*^2^*σ*) consists of two components. Conductivity can be written as Equation (8) [67]:

(8) $$\sigma =\int_{0}^{+\infty }\sigma \left(E\right)\left(-\frac{\partial f\left(E\right)}{\partial E}\right)dE,$$

where *σ*(*E*)—differential conductivity (the electrical conductivity of the electrons that fill the energy levels between *E* and *E* + d*E*), *f*(*E*)—the Fermi distribution function.

Then, thermo-emf *S* is determined by Mott’s Equation (9):

(9) $$S=\frac{1}{e\sigma T}\int_{0}^{+\infty }\sigma \left(E\right)\left(E-E_{F}\right)\left(\frac{\partial f\left(E\right)}{\partial E}\right)dE,$$

where *E_F_*—Fermi energy, *e*—elementary charge.

Thus, both components of power factor, Equations (8) and (9), are governed by differential conductivity σ(*E*) and by differential mobility *µ*(*E*): σ(*E*) is evaluated through density of charge carriers *n*(*E*) via density of states calculation (DOS) *g*(*E*); *µ*(*E*) depends on relaxation time *τ*(*E*) and effective mass m* (Equations (10–12)):

(10) σ(E)=n(E)eμ(E),

(11) n(E)=g(E)f(E),

(12) $$\mu \left(E\right)=e\frac{\tau \left(E\right)}{m^{*}},$$

The power factor is given by the following expression (Equation (13)):

(13) $$S^{2}\sigma =\frac{1}{e^{2}T^{2}}\frac{{\left(\int_{0}^{+\infty }n\left(E\right)e\mu \left(E\right)\left(E-E_{F}\right)\left(\frac{\partial f\left(E\right)}{\partial E}\right)dE\right)}^{2}}{\int_{0}^{+\infty }n\left(E\right)e\mu \left(E\right)\left(-\frac{\partial f\left(E\right)}{\partial E}\right)dE},$$

As it comes from Equation (13), the higher charge carriers’ concentration the higher power factor that is applied in n-type semiconductors (e.g., via alloying till their degeneration). In metals and degenerate semiconductors, Fermi statistics can be considered degenerate and expression for conductivity (Equation (8)) is simplified to a “metal” one and Mott’s equation (Equation (9)) becomes more ordinary “metal-like” (Equations (14) and (15)) [67]:

(14) $$\sigma =\sigma \left(E_{F}\right)=n\left(E_{F}\right)e\mu \left(E_{F}\right),$$

(15) $$S=\frac{\pi^{2}k_{B}{}^{2}}{3e}T{\left\{\frac{dln\sigma \left(E\right)}{dE}\right\}}_{E=E_{F}}=\frac{\pi^{2}k_{B}{}^{2}}{3e}T{\left\{\frac{1}{n}\frac{dn\left(E\right)}{dE}+\frac{1}{\mu }\frac{d\mu \left(E\right)}{dE}\right\}}_{E=E_{F}},$$

Although Equations (14) and (15) correspond to the limit case, they fit well to demonstrate the influence of such factors as charge carrier concentration *n*(*E*) and differential mobility *µ*(*E*) on thermoelectric properties of the material. As it follows from Equation (14), higher concentration *n* and mobility *μ* of charge carriers increases their conductivity *σ*, while the Seebeck’s effect (Equation (15)) is governed by their differential characteristics. Let us consider two routes to change thermo-emf in more detail [68].

**Figure 2:**
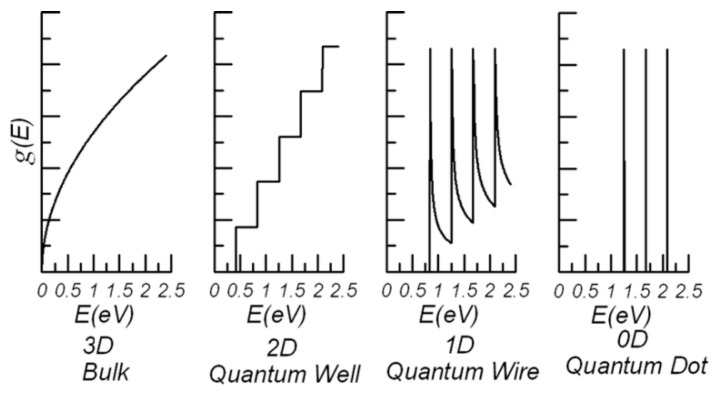
General behavior of the DOS function in the systems of different dimensionality. Reproduced with permission from Ref. [68]. Copyright 2005, *Acta Physica Polonica A*: Institute of Physics, Polish Academy of Sciences.

The first term in Equation (15) is *dn*(*E*)/*dE*, which is determined by DOS function behavior (Equation (11)). Figure 2 shows the general behavior of the DOS function [68].

Contrary to 3D systems, *dn*(*E*)/*dE* may take high values in the systems with less degrees of freedom due to the size-quantization effect on *g*(*E*). That is why nanostructured materials may exhibit higher *ZT* values, because electron gas can be confined there. Obviously, the phonon thermal conductivity becomes reduced in low-dimension systems due to intensive dissipation on the boundaries. Concerning practical applications, low-dimension systems should be prepared via nanoengineering methods in the volume of 3D thermoelectric material. Therefore, the same boundaries play a positive role as phonon dissipation sites to reduce thermal conductivity and size of the bulk thermoelectric or even may act as phonon filters according to the concept presented above.

The second term in Equation (15) is *dμ*(*E*)/*dE*, which is determined by behavior or time relaxation function *τ*(*E*) (Equation (12)). In other words, to increase thermo-emf one needs to achieve strong dependence of electrons dissipation on their energy, i.e., filtration of electron with respect to energy. The paper [68] referred to several examples of such filtration with different efficiency, however, the present review addresses the latest theoretical work [69]. It treats 2DEG as an energy barrier for electrons from the bulk on the grain boundaries. When calculating the thermoelectric characteristics of the material, the lower limit in the energy integrals in Equations (14) and (15) corresponds to this energy barrier. Such approach is quite inaccurate, because it is based on classical physics and does consider electron tunneling through the barrier. Anyway, it demonstrates qualitatively that filtration of bulk electrons amplifies *ZT* by a factor of 1.5 at optimal barrier height, which slightly exceeds the Fermi level. This agrees well with the point stating the significance of *τ*(*E*) dependence in the region of Fermi level according to the metal-like Equation (15) for *S*.

To summarize the theoretical aspects of thermoelectricity, nanostructured materials should possess enhanced thermoelectric characteristics. The diversified net of mutually connected boundaries in the bulk nano-thermoelectric can serve as phonon filters (important for low temperatures) or simply as sites for phonon dissipation. The 2DEG state appearing there can act as electron filters to improve thermoelectric characteristics of the bulk materials as well as form an individual low-dimension phase with enhanced thermoelectric properties improving the overall material’s characteristics.

## 4. Thermoelectricity on Oxides Interfaces

### 4.1. Oxide Heterointerfaces

**Figure 3:**
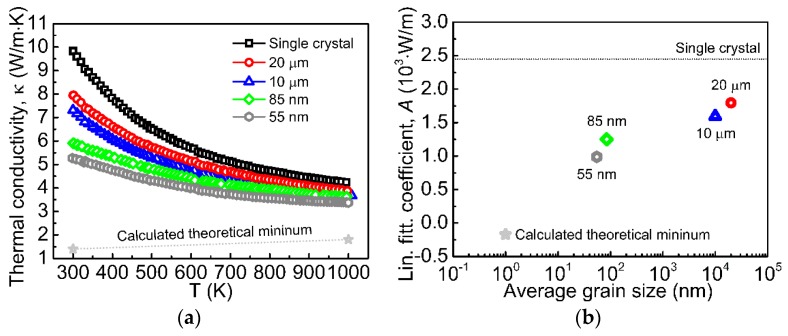
Temperature dependence (**a**) of effective thermal conductivity for SrTiO_3_ polycrystalline ceramics and single crystal (also shows a calculated theoretical minimum for disordered crystals with few nm grain size). Size dependence (**b**) of linear coefficient *A* for fitting *κ*(*T*) = *κ*_o_ + *A*/*T* + *B*/*T*^2^. Estimated average grain sizes are shown for some representative samples according to Reference [10].

As shown in Section 3, multiple boundaries in the bulk thermoelectric can play a positive role in increasing phonon dissipation and decreasing thermal conductivity, as demonstrated by K. Koumoto et al. for SrTiO_3_ [10] (Figure 3). Additionally, there may be formed regions of 2DEG localization on the grain boundaries of the oxide materials.

A striking example is the LaAlO_3_/SrTiO_3_ system, for which A. Ohtomo et al. showed 2DEG formation with high electron mobility [70]. It arises due to polarity discontinuity between alternating polar lattice (AlO_2_)^−^/(LaO)^+^ and neutral one (TiO_2_)^0^/(SrO)^0^. Electron mobility in the interface (LaO)^+^/(TiO_2_)^o^ appeared rather high for observing dramatic periodic oscillations of magnetoresistance at low temperatures (2–5 K) in response to magnetic field inversion, which indicates quantum transport. N. Reyren et al. demonstrated 2D superconductivity for that 2DEG with T_s_ = 0.2 K in 2007 [71]. And finally, in 2016, I. Pallecchi et al. revealed a large contribution of phonon drag to thermo-emf when studying the thermoelectric properties of the LaAlO_3_/SrTiO_3_ boundary at low temperatures (<50 K, peak at ~12–14 K) [72]. Formation of 2DEG via the similar mechanism on the hetero interface (LaO)^+^/(TiO_2_)^0^ was shown for the LaGaO_3_/SrTiO_3_ system [73].

The mechanism is a rather universal one for complex perovskite-type oxides with the general formula ABO_3_ (where A—alkali-earth or rare-earth metal, B—transition 3d, 4d or 5d metal) [74]. As an example, let us consider RTiO_3_/SrTiO_3_-type hetero interfaces (where R—rare earth metal) [75,76,77,78,79]. There are alternating atomic layers RO/TiO_2_/SrO on the boundary. Titanium ions in the middle layer (TiO_2_) exhibit a “struggle” between oxidation states +3 and +4. In the sequence of (Ti^4+^O_2_^4−^)^0^/(Sr^2+^O^4−^)^0^ layers of SrTiO_3_ structure, TiO_2_ layer should be neutral, while in the sequence of (R^3+^O^2−^)^+1^/(Ti^3+^O_2_^4−^)^−1^ layers it should be charged. As a result, (R^3+^O^2−^)^+1^ layer acts as donor of electrons for the TiO_2_ layer leading to the 2DEG formation on it. From another point of view, when R acts as alloying agent for SrTiO_3_, but alloying occurs only along phase boundary. Beyond that, as in ordinary alloying, R acts as an electron donor. Another feature of such “planar” alloying is the absence of alloying agent in the region of 2DEG localization, which leads to absence of dissipation of electrons on R+ ions in the crystal lattice (this effect cannot be achieved for 3DEG at bulk material alloying). It is noteworthy that additional alloying of SrTiO_3_ bulk phase with reasonable amount of electron donors does not lead to its spontaneous polarization, while additional electrons attracted by arbitrarily positive (RO)^+1^ layer on the grain boundary should increase the density of 2DEG [77].

Obviously, in more general case the contact between polar and nonpolar materials is not obligatory, but the main point is the unbalanced polarizations between the layers on the boundary. For example, Mg_x_Zn_1−x_O/ZnO hetero structure consisting of piezoelectric materials exhibit 2DEG formation on the boundary, because the piezo-effect (caused by stress due to different lattice parameters) leads to unbalanced polarization and 2DEG formation [80,81,82,83]. This allowed the observation, for the first time, of the quantum Hall effect in the oxide system based on Mg_0.15_Zn_0.85_O/ZnO [80] in 2007, and in 2010, A. Tsukazaki et al. demonstrated a fractional quantum Hall effect [81].

**Figure 4:**
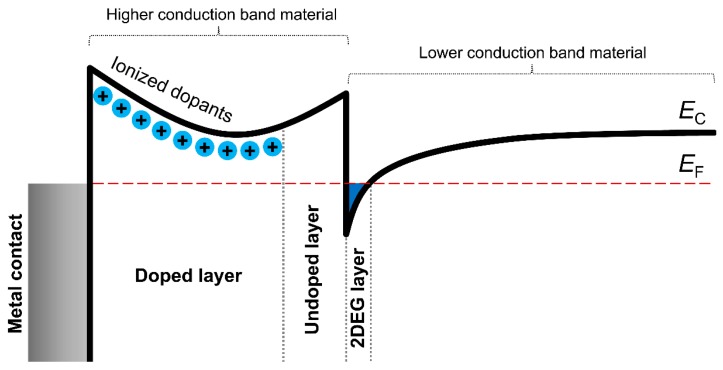
Principle of modulation doping to obtain a high-mobility 2DEG according Ref. [74].

Another mechanism of 2DEG formation on the hetero interface of oxide materials is modulation doping [74]. Electrons from the n-doped material tend to leave it and to occupy the second material at the contact, because the conduction band edge of the first material is higher than in the second one (Figure 4). At the same time, there occurs a non-compensated bulk charge in the first material that distorts the conduction bands locally in the region of the contact with the second material. This yield in electron trap occurring in undoped material near the interface boundary and it is thin enough to form 2DEG. The undoped layer of the material with higher conduction band edge enhances this effect (Figure 4) [74].

Modulation doping is fairly known and used in heterostructures of AlGaAs/GaAs [84,85,86,87,88]. But that approach is suitable for oxide hetero interfaces. For example, S. Stemmer and S. James Allen suggested considering perovskite hetero interfaces SrTiO_3_/LaAlO_3_ and SrTiO_3_/SrZrO_3_, because the conduction band edge of SrTiO_3_ [89,90,91,92] is lower than that for LaAlO_3_ and SrZrO_3_ [93,94,95]. Formation of 2DEG on the hetero interface of SrTiO_3_ and n-doped SrZrO_3_ perovskites are proved by the results of numerical modeling [96] and by modulation doping experiments for SrTiO_3_/Sr(Ti,Zr)O_3_ [97]. 2DEG formation was also demonstrated for the hetero interface of structurally different oxides, TiO_2_/SrTiO_3_ [27].

Recently, Jun-ichi Fujisawa et al. evaluated conduction-band edges for these oxides and showed that the conduction-band edge of SrTiO_3_ is 0.40 eV higher than for TiO_2_ (i.e., modulation doping of TiO_2_/SrTiO_3_ hetero interface is plausible) [98]. It is noteworthy that there were studies on undoped TiO_2_ grown on SrTiO_3_ [27]. The authors claimed that TiO_2_ epitaxial films formed by pulsed laser deposition (PLD) may lack oxygen, which is extracted from SrTiO_3_ yielding the TiO_2_/SrTiO_3−δ_ hetero interface. It is known for SrTiO_3_ that oxygen-deficiency is equivalent in some way to substituting Sr with La [99]. Therefore, it can be concluded that 2DEG formation on the TiO_2_/SrTiO_3_ hetero interface described in [27] is caused by modulation doping.

### 4.2. Thermoelectric Properties of Superlattices and Heterointerfaces Based on SrTiO_3_ Oxide

Let us consider several important works regarding the theme of our review followed by the idea explanation. First of all, let us pay attention to [100], which studies thermoelectric properties of superlattices based on SrTiO_3−δ_, fabricated using PLD. The superlattices consisted of pairs of layers with 5 at.% of Sr being substituted with Pr ((Sr_0.95_Pr_0.05_)TiO_3_) and 20 at.% Ti ions being substituted with Nb (Sr(Ti_0.8_Nb_0.2_)O_3_). The whole series of the studied systems can be presented as [(Sr_0.95_Pr_0.05_)TiO_3−δ_]_x_/[Sr(Ti_0.8_Nb_0.2_)O_3−δ_]_y_ for pairs of (x, y) ∈ {(x, 0); (8, 2); (8, 5); (8, 8); (5, 8); (2, 8); (0, y)}. Superlattices were composed of 20 pairs of layers (x,y) with the layer thickness for determination of their individual characteristics ((x, 0) and (0, y)) being ~500 nm (at least for thermal conductivity measurements).

Temperature dependence on planar conductivity *σ* [100] and Seebeck’s coefficient *S* for superlattices are not somewhat extraordinary (obviously, also the power factor *S*^2^*σ*) and change gradually from individual characteristics of two types (from (Sr_0.95_Pr_0.05_)TiO_3_ to Sr(Ti_0.8_Nb_0.2_)O_3_ and vice versa). Thus, dependences can be described by the model of parallel arranging of independent layers. That means formation of 2DEG was not observed in the studied systems, which will be explained later in the review.

Unfortunately, paper [100] did not include direct temperature dependences of planar thermal conductivity *κ*. Only *κ*(*T*) was found in the transverse direction separately for (Sr_0.95_Pr_0.05_)TiO_3_ and Sr(Ti_0.8_Nb_0.2_)O_3_ layers of large thickness ~500 nm. Comparison was done for layers’ thickness ranging from ~0.8 nm for 2 unit cell to ~3.1 nm for 8 unit cell, which is assumed to be equal in thermal conductivity in planar direction due to the isotropic character of the materials properties with cubic crystal structure. For superlattices, temperature dependence *κ* in planar direction was assessed within the model of parallel thermoresistance. The authors in [100] admit that real thermal conductivity have to be lower mainly due to additional dissipation on hetero interfaces.

Based on obtained *κ* assessment and measured values of *σ* and *S*, the work [100] also provides an evaluation of the *ZT* value. It was unexpectedly found that maximal temperature characteristic with respect to the *ZT* value was shown by superlattice (8, 8) with 0.11 value at 300 K and 0.46 at 1000 K. Because thermal conductivity value was overestimated, the *ZT* value is underestimated. The final conclusion on true values can be done via direct measurements of *κ*(*T*) in planar direction for such superlattices. Anyway, it is revealing that bicomponent system without specific 2D effects can be improved regarding the *ZT* value via optimal adjustment of components ratio with the optimum being at 1:1 ratio.

**Figure 5:**
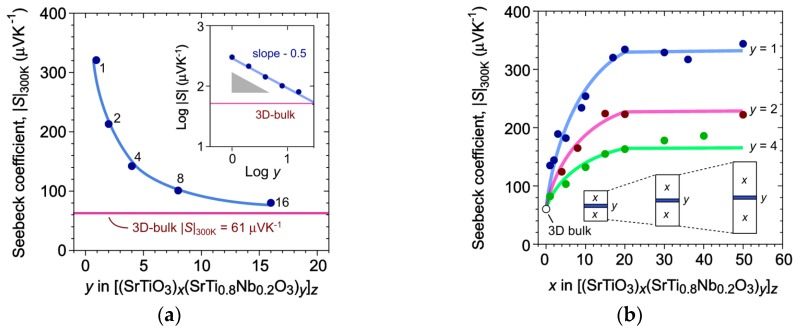
Thermo-emf dependence of Sr(Ti_0.8_Nb_0.2_)O_3_ of layer at 300 K for [SrTiO_3_]_x_/[Sr(Ti_0.8_Nb_0.2_)O_3_]_y_ superlattice: (**a**) On y (thickness of the conducting layer) at sufficient thickness of insulating layers; (**b**) on x (thickness of insulating layers) at sufficient thickness of conducting layers. Reproduced from [101].

There is a series of works that studied the influence of component ratio in the superlattice on its thermoelectric characteristics [27,101,102]. [SrTiO_3_]_x_/[Sr(Ti_0.8_Nb_0.2_)O_3_]_y_ systems were studied with x = 1–60 and y = 1–20 for 20, 24, and 100 pairs of layers fabricated also using the PLD method. Thermo-emf (Seebeck’s coefficient) *S* in the planar direction was chosen as a key thermoelectric characteristic for these superlattices. *S* value was shown to be 320 μV/K at 300 K (5 times higher than 61 μV/K achieved for bulk Sr(Ti_0.8_Nb_0.2_)O_3_) for 1 unit cell layers of degenerate insulator Sr(Ti_0.8_Nb_0.2_)O_3_ isolated by rather thick (>16 unit cells) layers of nondegenerate SrTiO_3_ insulator. The thermo-emf of Sr(Ti_0.8_Nb_0.2_)O_3_ conducting layer is inversely proportional to the square of its thickness (Figure 5a), as it should be for 2DEG [52]. When thickness reaches 16 unit cell, *S* value is nearly the same as for the bulk material that can be interpreted as 2DEG failure. 2DEG was also shown to be affected by the thickness of SrTiO_3_ insulating layer (Figure 5b). The *S* value of the conducting Sr(Ti_0.8_Nb_0.2_)O_3_ of variable thickness is observed to decrease when the thickness of insulating SrTiO_3_ is below 6.25 nm (16 unit cells) reaching the value or the bulk Sr(Ti_0.8_Nb_0.2_)O_3_ at 0 unit cell of SrTiO_3_.

In the first case, electron gas localization and 2DEG formation is caused directly by the thickness of the conducting layer, i.e., confinement of the electrons space. In the second case the situation is a bit more complicated. Strict localization of the electron gas in the Sr(Ti_0.8_Nb_0.2_)O_3_ layer fails due to electron tunneling through the SrTiO_3_ insulating layer, namely, the thinner SrTiO_3_ the less localized electrons become in the Sr(Ti_0.8_Nb_0.2_)O_3_ layer leading to gradual failure of 2DEG state.

Taking into account the results of [27,101,102] it is clear why authors in [100] did not observe 2DEG formation. Both systems are similar in some way, because in both cases good conducting Sr(Ti_0.8_Nb_0.2_)O_3_ layers alternate with insulating ones. Thus, in [100], conductivity of (Sr_0.95_Pr_0.05_)TiO_3_ was only 18 S/cm at 300 K as compared to 3504 S/cm for Sr(Ti_0.8_Nb_0.2_)O_3_, while the best candidate for 2DEG among the considered superlattices is the [(Sr_0.95_Pr_0.05_)TiO_3-δ_]_8_/[Sr(Ti_0.8_Nb_0.2_)O_3−δ_]_2_ system. According to [27,101,102], 2 unit cells thickness of the conducting layer drastically weakens 2DEG, the same does 8 unit cells of insulating layer causing delocalization of electron gas due to tunneling. Additionally, (Sr_0.95_Pr_0.05_)TiO_3_, unlike insulating SrTiO_3_, is a degenerate semiconductor, which also complicates the situation. In conclusion, 2DEG did not form in [100] due to electron gas that was not localized enough or its formation was of a minor nature for a clear identification.

Unfortunately, the series of works [27,101,102] did not provide direct thermal and electric conductivity measurements. The *ZT* value was assessed via evaluation at 300 K using Seebeck’s coefficient S, thermal conductivity for SrTiO_3_ single crystal, and conductivity *σ* calculated from Equation (14) with the measured charge carriers concentration and reference data on their mobility. The *ZT* value for 2DEG at 300 K was found to be 2.4—that is extremely large. Mean *ZT* for the superlattice was 0.24, which is nearly twice as large than the *ZT* value (0.11 at 300 K) for superlattice (8,8) from [100]. This mainly proves the advantage of the 2DEG-based strategy for the SrTiO_3_-containing systems over the conventional doping and over the simple combination of differently alloyed thermoelectric materials.

The paper [27] also studied the properties of a TiO_2_/SrTiO_3_ hetero interface formed on the boundary of a 56 nm TiO_2_ layer and a 0.5 mm SrTiO_3_ layer. For that system, the depth profile of carrier concentration was obtained. There is a peak observed in the region of ~0.3 nm-thick of TiO_2_ side in the heterointerface, which exceeds by 2–2.5 orders of the magnitude the charge carriers concentration in the bulk of TiO_2_. This clearly proves the spontaneous 2DEG formation on the heterointerface of such oxides due to the reasons described in the previous section. Thermo-emf *S* and conductivity *σ* were measured for the TiO_2_/SrTiO_3_ heterointerface, SrTiO_3_ after mechanical removal of TiO_2_ layer, and for a separate 126 nm-thick TiO_2_ layer [27]. Thermo-emf *S* of TiO_2_/SrTiO_3_ was shown to be 1050 μV/K at 300 K and is caused mainly by the presence of heterointerface. Unfortunately, any assessments of the *ZT* value for that heterointerface were absent.

Based on results from [27,101,102], papers [10,11,69] presented the design of a hypothetic material, which can efficiently utilize 2DEG properties, and modelling the properties of such material. It was proposed to fabricate the SrTiO_3_-based material doped with ions in both sublattices. Particularly, grains of (Sr,La)TiO_3_ containing 2–7 at.% of La^3+^ in the positions of Sr^2+^ are supposed to be separated by thin layers (called “grain boundaries” in these papers) of Sr(Ti,Nb)O_3_ containing 20 at.% of Nb^3+^ in the Ti^3+^ positions. Both scenarios of substitution lead to free electrons occurring in the structure, while charge compensation is provided by oxidation state change of titanium from +4 to +3 (Ti^4+^ + e^−^ → Ti^3+^). Substituting Ti with Nb leads to decrease of the conduction band edge, while Sr substitution with La has no effect on it, thus, causing 2DEG formation on the grain boundaries [10,11,69,99].

**Figure 6:**
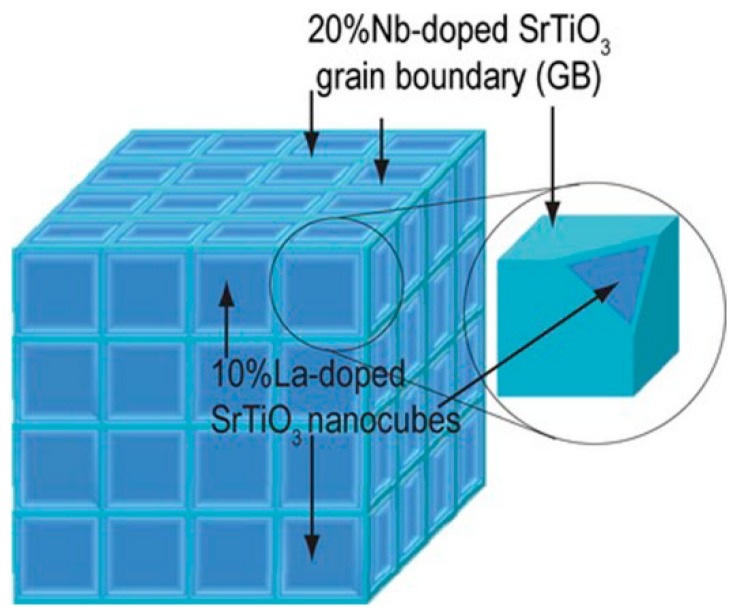
“Rubik’s cube” structure of the SrTiO_3_ ceramics (2DEG grain boundaries are shown in light blue, grain interiors—in deep blue). Reproduced with permission from Reference [11].

In the suggested system, grains are in cubic modification [10,11,69]. Therefore, the whole system is a superposition of three [(Sr_1−a_La_a_)TiO_3_]_x_/[Sr(Ti_0.8_Nb_0.2_)O_3_]_y_ superlattices along three mutually perpendicular dimensions (Figure 6). The superlattice itself along one of the dimensions can be considered as an analogue to the superlattices studied in [27,101,102], while the La doping controls the total electron concentration in the system. That is why, the size of cubic grains in papers [10,11,69] was chosen to be 16 unit cells (x = 16) in accordance with [27,101,102]. On one hand, such geometry is necessary to prevent the collapse of electron confinement, but on the other, to maximize 2DEG concentrated on the boundaries with respect to the total volume of the system. Thickness of well-conducting Nd-doped grain boundaries ranged from 1 to 16 unit cells.

When calculating thermoelectric characteristics of the material (Figure 6), the authors in [10,11,69] used thermoelectric properties of the boundaries from [27,101,102], while thermoelectric properties of grains were modelled given 2DEG occurs on their boundaries. For electrons in the bulk of grains, 2DEG was considered as a charged shell acting as a potential barrier for free electron motion in the material’s bulk or, in another words, it behaves as an “electron filter” (See Section 3). The *ZT* value for such material with minimal thickness of Sr(Ti_0.8_Nb_0.2_)O_3_ boundaries (1 unit cell) was shown to be ~0.8–1.2 at 300 K depending on the height of potential barrier created by 2DEG.

Some criticism on the works [10,11,69] is provided in Section 5. After all, the results by K. Koumoto et al. clearly demonstrate the nanostructuring as the key approach to drastic improvement of the oxide’s thermoelectric characteristics.

## 5. SrTiO_3_-based Biphase Ceramics

### 5.1. Some Experience on Manufacturing of the “Biphase” Thermoelectric Materials

Epitaxial growth of superlattices or their fabrication by other methods enabling precise phase arrangement, all these techniques are “lab-scale” and not suitable for large-scale production. Even for epitaxial methods, the fabrication of a non-planar 3D superlattice similar to the on discussed in [69] is challenging. This suggests to us to turn an eye on less “sophisticated” approaches of materials engineering with the corresponding experimental results being discussed below.

Primarily, let us pay attention to the papers [103,104], which studied the fabrication of La-doped SrTiO_3_ nanocubes with Nb-doped surface. It may be considered as an attempt to prepare structures of the type investigated in [69]. Such nanocubes were synthesized via a rapid synthesis combining a rapid sol-precipitation and hydrothermal process. The La-doped SrTiO_3_ nanocubes were formed at room temperature by a rapid dissolution of NaOH pellets during the rapid sol-precipitation process, and the Nb-doped surface (shell) along with Nb-rich edges formed on the core nanocubes via the hydrothermal process. Size of the obtained cubes ranges from 80 to 150 nm [104] with the thickness of Nb-doped layer being 3–4 nm [103]. The paper [104] also features self-assembling of such “cells” at slow drying on SiO_2_/Si support. Cubes tend to form dense packed face-to-face clusters forming a continuous smooth layer of 10 µm thickness. Unfortunately, the consolidation of such a system was not studied.

The best characteristics in the theoretical paper [69] was found for a structure with the following properties: Boundary thickness of 1 unit cell (~0.3 nm) to form high density 2DEG; grain size of 16 unit cells (~6.5 nm) to prevent tunneling of electrons localized on grain boundaries as 2DEG. This is in agreement with earlier results presented in [102]. It is noteworthy that thickness of Nb-doped surface of La-doped SrTiO_3_ nanocubes is very high [103,104]. Even at ideal consolidation two such cubes form compound Nb-doped layer ~7 nm thick that is already large and leads to complete 2DEG failure [102]. Size of such nanocubes is, of course, high enough to isolate reliably the intergranular layers from each other. However, at that size the contribution of intergranular phase to the total thermoelectric characteristics appears to be rather small, even in case of its thickness small enough for 2DEG formation. Thus, nanocube system presented in [103,104] can provide only an advantage of electron filtration. Apparently, further technological advances towards fabrication of such cubes will finally allow obtaining small enough La-doped SrTiO_3_ nanocubes with rather thin Nb-doped surface to implement hypothetical system presented in [69].

Another attempt to implement hypothetical nanostructured material from [69] is described in [105], where design of La-doped SrTiO_3_ grains with Nb-doped surface was done via an alternative route. As a starting material the commercial 5 at.% La-doped SrTiO_3_ (Titan Kogyo Co., Tokyo, Japan) was used. Surface layer of NbO_x_ was formed on powder particles via adding Nb(OEt)_5_ oxalic acid aqueous solution followed by filtration and drying. Modified and unmodified powders were sintered using spark plasma sintering at 950 °C in ~3 min at 200 MPa. Sintering yielded in fine-grained ceramics containing dense-packed particles with identical size distribution ranging in 200–500 nm. This work demonstrated two main features, namely, NbO_x_ does not form separate phase of non-perovskite type but is chemically bound to main SrTiO_3_ material. Additionally, Nb localization in the system has been addressed.

Nb concentration in Ti positions was found to be 1.3 ± 0.4 at.% [105]. It was demonstrated that unlike doping with additional element and fairly close charge carrier concentrations, modified sample is a worse conductor than unmodified one. The authors explains this result with space localization of Nb along grain boundaries.

Substitution of Ti with Nb in SrTiO_3_ decreases conduction band edge [105]. Thus, there is formed a zone of potential minimum for electrons along the boundaries and charge concentrating occurs. Charged grain boundaries play a role of potential barrier for conduction band electrons, acting as an electron filter in accordance with concepts from [69]. Such concepts do fit well to explain lower conductivity of the modified sample (part of the electrons does not pass through the potential barrier on the grain boundaries) as well as higher thermo-emf (electron filter in Equation (15)) and power factor. Unfortunately, space localization of Nb or charge on the boundary were not found directly in [105]. Meanwhile, thickness of conductive layer even at 6–7 nm completely violates electron confinement with 2DEG state being collapsed [102]. Additionally, grain size of ceramics obtained in [105] ranges from 100 to 600 nm, which is too large to observe any valuable contribution of 2DEG to thermoelectric properties of the material, as indicated by authors.

According to theoretical paper [69], at optimal barrier height the power factor of the main material (without taking into account thermoelectric characteristics of the boundaries) can increase by ~36%. Power factor was observed to increase by the same order of magnitude in paper [105] (~35%) when modified and unmodified ceramics were compared at optimal conditions (450 K). This is the strongest argument for Nb and charge localization along the boundaries. However, there is no reasons to suppose that electrons near the boundaries are in the 2DEG state. On the one hand, thickness of NbO_x_ layer applied on the powder particles is unknown, on the other hand, thermal diffusion during sintering may have led to Nb-rich zone, too wide for 2DEG formation, while properties enhancement occurred due to electron filtration and increased total number of electrons in the system. Additionally, the authors in [105] did not consider influence of heterophase boundaries between the grains on thermal conductivity. Obviously, such boundaries may efficiently dissipate phonons that contributes to reduction of thermal conductivity and increases the *ZT* value.

It can be temporary summarized that purely ceramics approach for systems with electrons concentrated along grain boundaries is one of the most prospective. The key problem towards its successful application consists in creating an electron-scavenging layer. That is why, in our opinion, it is reasonable to shift towards biphase ceramics, in which 2DEG is spontaneously formed on the boundary between two phases. Some variants of heterointerfaces providing 2DEG have already been discussed earlier here. Let us consider some complications regarding preparation of such structures in the form of ceramics in more detail.

Sintering of biphase ceramics from two structurally close materials can involve mutual thermal diffusion of the elements. It easy to demonstrate for the grain boundaries of SrTiO_3_/GdTiO_3_. Heterointerface itself can be considered as Gd-doped SrTiO_3_ layer of 1 unit cell thickness. Because SrTiO_3_ is easily doped with Gd [61], the problem of single-phase formation is rather topical here. But even for that case thermal diffusion at sintering may initiate Gd doping of SrTiO_3_ grains surface. This will yield in spreading Gd-doped layer, expanding the region of electron gas localization, and, as a result, collapsing 2DEG. Paper [75] showed for GdTiO_3_/SrTiO_3_ layers grown by molecular beam epitaxy that electron gas density reduces by 20% after the introduction of Sr ions. However, at Sr concentration >4 at.%, Gd_1−x_Sr_x_TiO_3_ undergoes insulator-to-metal transition, which is a feature of rare earth titanates [106]. Therefore, surface alloying of GdTiO_3_ ceramics grains by Sr at thermal diffusion may also lead to expansion of electron gas localization area and 2DEG collapse.

**Figure 7:**
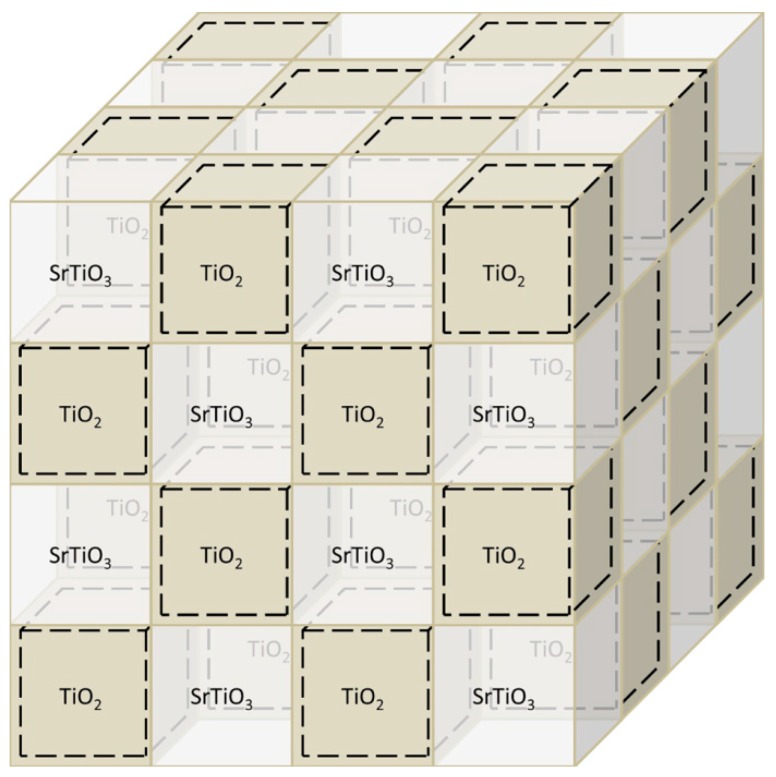
Ideal structure of TiO_2_−SrTiO_3_ biphase ceramics.

Thus, mutual atom diffusion at sintering of structurally close compounds may give a rise to a number of complications yielding a 2DEG collapse due to delocalization of conduction band along grain boundaries. That is why we suggest to utilize the effect of spontaneous 2DEG formation on the interface of two different phases, e.g., TiO_2_/SrTiO_3_ [27]. TiO_2_ and SrTiO_3_ will hardly form single phase, while 2DEG is obtained on their heterointerface because of modulation alloying owing to difference in conduction band edge [98], which is hardly affected by La-doping [99]. That is why, even mutual exchange of alloying elements may unlikely lead to collapse of 2DEG state. Thus, unlike theoretical paper [69], we suggest a biphase structure composed of TiO_2_ and SrTiO_3_ grains (Figure 7). The dashed area shows 2DEG localization from the TiO_2_ side in the interface. Relative scale corresponds to 1 unit cell of TiO_2_ localizing 2DEG and to 16 unit cells of TiO_2_, which are necessary to prevent electron tunneling (Figure 7).

As was mentioned above, the *ZT* value of the hypothetic model SrTiO_3_-based material utilizing 2DEG properties at most reaches 0.8–1.2 at 300 °C at minimal thickness of separating layer and small grain size [69]. These factors enable one to expect drastic improvements of thermoelectric properties of the suggested TiO_2_–SrTiO_3_ biphase ceramics (Figure 7). It is worth mentioning again that electron tunneling through the thin (1 unit cell) charged edge with 2DEG was not taken into account in [69]. For real systems, this may lower the effect of electron filtration and reduce thermoelectric efficiency.

On the other hand, structure of real ceramic systems will be quite different from the ideal model (Figure 7). However, if TiO_2_ and SrTiO_3_ grains are in checkered order in the bulk, then 2DEG localized on the heterointerface of these phases will form the integrated network along the surface between the grains permeating throughout the whole material.

The density of a real 2DEG network can be lower than in the ideal system, because grain shape in perovskite ceramics is usually a truncated cube rather than ideal cube as considered in the model. Anyway, grain size plays the major role and is hard to retain within the range found for the ideal system. Another hurdle is the fabrication of highly dense ceramics with minimal variance of grain size, i.e., it is undesirable to obtain lower grain sizes than is required to separate reliably different 2DEG regions from each other and to prevent 2DEG collapse. At this regions, 2DEG may be partially or completely collapsed leading to decline in performance of the whole 2DEG network. Also, small grain size is not only necessary for increasing of 2DEG contribution to the thermoelectric characteristics of the material. Transfer from coarse-grained to fine-grained ceramics allows the improvement of the physico-chemical properties of the material (hardness, fracture viscosity, ductility and etc.) due to significant contribution of intergranular boundaries states [107]. Fabrication of highly-dense biphase ceramics and its challenges will be discussed in the next section.

Another important feature in the real ceramics system is the additional loss of electron energy in 2DEG network at the curved places of connection between separate 2DEG regions on the grain boundaries. 2DEG transport along the grain boundaries is similar to transport in superlattice within the context of ideal system, because various 2DEG planes in the network are mutually orthogonal [69]. However, in real system separate 2DEG surfaces on grain boundaries may be curved and are arranged chaotically to each other. That is why it is impossible to choose some direction, along which the network would perform the same as the superlattice. Meanwhile, it was shown for the similar problem of electron motion in deformed graphene layer that in the curved places additional dissipation of motion energy occurs [108,109]. Similar processes should also take place in the 2DEG network of the real ceramics that can have a negative impact on its thermoelectric properties as compared to an ideal system.

Indicated issues clearly show that *ZT* values reported in [69] for an ideal system are rather overestimated. But in general, TiO_2_–SrTiO_3_ biphase ceramics with ordered 2D surface along grain boundaries should be characterized by enhanced thermoelectric properties due to 2DEG formation on this surface.

**Figure 8:**
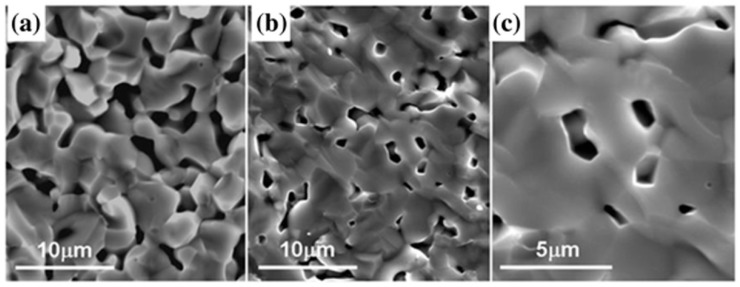
SEM images of Nb−SrTiO_3_ ceramics (**a**) with and (**b**,**c**) without the surface modification of nanosized titania. © 2016 Li E, Wang N, He H, Chen H. Published in [110] under CC BY 4.0 license. Available from: https://doi.org/10.1186/s11671-016-1407-8.

**Figure 9:**
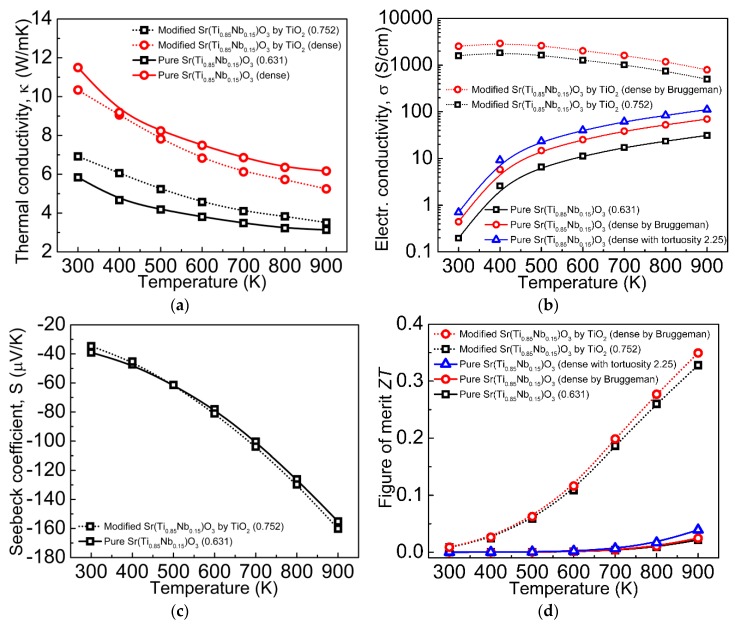
Temperature-dependent thermoelectric properties of Nb−SrTiO_3_ ceramics obtained from submicron Sr(Ti_0.85_Nb_0.15_)O_3_ powder either modified or non-modified with TiO_2_: (**a**) Thermal conductivity; (**b**) Electrical conductivity; (**c**) Seebeck coefficient; (**d**) Figure of merit *ZT*. © 2016 Li E, Wang N, He H, Chen H. Published in [110] under CC BY 4.0 license. Available from https://doi.org/10.1186/s11671-016-1407-8.

Let us consider experimental work devoted to SrTiO_3_-based ceramics with the addition of TiO_2_ [110]. Single-phase Nb-doped submicron powder of strontium titanate Sr(Ti_0.85_Nb_0.15_)O_3_ was prepared via solid phase synthesis from highly pure powders of SrCO_3_, TiO_2_, and Nb_2_O_5_ in argon atmosphere at 1400 °C for 4 h. Additional surface modification of the powder particles with nanosized particles of TiO_2_ was performed by liquid phase precipitation of (NH_4_)_2_TiF_6_ (0.06 M) and H_3_BO_3_ (0.2 M) in the water solution for 2 h at room temperature [111]. Pressed at 20 MPa samples of modified and nonmodified powders were being sintered in graphite crucible in argon atmosphere at 1500 °C for 3 h. Porous ceramics samples were obtained (Figure 8) with significantly different thermoelectric properties (Figure 9).

Because obtained thermoelectric materials are porous, the influence of porosity on thermoelectric properties should be addressed. Thermal conductivity of such media is usually described by P.G. Klemens (Equation (16)) [112]:

(16) $$\kappa_{eff}=\kappa_{o}\left(1-4p/3\right),$$

where *κ_eff_*—effective thermal conductivity of the porous medium; *κ*_o_—thermal conductivity of the full dense material; *ρ*—porosity.

Relative density of the original and titania-modified Nb−SrTiO_3_ samples are 63.1% and 75.2%, respectively. Due to the different porosity of the materials, the paper [110] compared their thermal conductivity with recounting according to P.G. Klemens’ Equation (16): Thermal conductivity of more porous nonmodified Nb−SrTiO_3_ sample was recounted at density of less porous titania-modified one. At the same time, Figure 9a shows thermal conductivity recounted by us in accordance with Equation (16) for full dense modified and nonmodified materials. As can be seen, surface modification of strontium titanate Sr(Ti_0.85_Nb_0.15_)O_3_ powder particles with titania greatly reduces thermal conductivity. Given the grain size for both ceramics is the same (order of few microns, Figure 8) and TiO_2_ content is small (not provided, but it can be concluded from the synthesis routine [110]), thermal conductivity reduction should be caused by high phonon dissipation on the heterophase interfaces. Additionally, this again proves the prospects of thermoelectrics based on TiO_2_−SrTiO_3_ biphase ceramics.

Conductivity of the two samples is also rather different (Figure 9b). The work [110] just notes surface modification of Sr(Ti_0.85_Nb_0.15_)O_3_ powder particles with titania drastically increases conductivity. The effect of porosity is not considered as in the case of thermal conductivity. The same problem of porosity effect on conductivity of oxide ceramics was studied in [113]. Although this paper [113] reported on the conductivity of nondegenerate insulator La_2_Mo_1.5_W_0.5_O_9_, while SrTi_0.85_Nb_0.15_O_3_ is a degenerate one in this case, approaches suggested they are of universal nature.

For biphase media, where one phase is incapsulated into the other one as spherical inclusions, authors in [113] suggest to use two models of conductivity: Maxwell−Garnett [114,115] and Bruggeman [116,117]. These models assume the conductivity of the incapsulated medium to be zero that corresponds to pores. There are Equations (17) and (18) in [113]:

(17) $$\sigma_{eff}=\sigma_{0}\frac{1-p}{1+p/2},$$

(18) $$\sigma_{eff}=\sigma_{0}\frac{2-3p}{2},$$

where *σ_eff_*—effective conductivity of the porous material; *σ*_o_—conductivity of the full dense material.

Equation (17) based on the Maxwell−Garnett model is valid till porosity 0.135, therefore, it is not applicable to materials from [110]. Equation (18) based on the Bruggeman model is valid till porosity values up to 0.22, although it was shown to be applicable till ~0.28 [113]. That is why we used Equation (18) to evaluate conductivity of full dense material from the data on more dense material obtained via surface modification of Sr(Ti_0.85_Nb_0.15_)O_3_ with titania [110]. A less dense sample obtained from powder without surface modification was also subjected to analysis. It was shown to implement tortuosity factor *τ* [118] for conductivity assessment of highly porous materials [113] in order to take into account complex geometry of porous channels (Equation (19)):

(19) $$\sigma_{eff}=\sigma_{0}\frac{1-p}{\tau },$$

To determine tortuosity one needs more extensive experimental data like the one presented in [113]. Particularly, for highly porous system with porosity *Р* = 0.34 the tortuosity was found to be 2.25 [113]. Given this, we used tortuosity *τ* = 2.25 for more correct assessment of conductivity of less dense material (Equation (19)) obtained from Sr(Ti_0.85_Nb_0.15_)O_3_ without modification with titania [110]. We did not take into account pore geometry, which can be different for the ceramics studied in [110] and [113].

Figure 9b provides conductivity values calculated using Equations (18) and (19) for full dense materials, modified and nonmodified ones. As can be seen, addressing porosity shortens the difference in conductivity between the materials a bit, although the gap is still of several orders of magnitude.

Temperature characteristics of Seebeck’s coefficient *S* for both materials are very close [110] (Figure 9c). Effect of porosity on *S* still remains unclear [119]. On one hand, pores are equivalent to removing a part of thermoelectric material from the volume, therefore, it should proportionally reduce the *S* value. On the other hand, pores may act as dissipation sites for low electrons [120], performing as electron filters and having a positive impact on the *S* value. In general, temperature dependence data on *S* from [110] is close to the one obtained for dense Sr(Ti_0.8_Nb_0.2_)O_3_ ceramics from [121] (Figure 9c).

The paper [110] reported *ZT* values calculated for the data without addressing porosity and it turned out to be drastically different for Sr(Ti_0.85_Nb_0.15_)O_3_ ceramics and for the one modified with titania (Figure 9d). We have done revision using corrected values, but it did not make much difference for temperature dependence of the *ZT* value (Figure 9d). Apparently, corrections for thermal and electrical conductivity are of the same order and compensate each other at evaluation of the *ZT* value, because *ZT*~*σ*/*κ*. Temperature dependence of *ZT* value for titania-modifed Sr(Ti_0.85_Nb_0.15_)O_3_ is close to the one evaluated for Sr(Ti_0.8_Nb_0.2_)O_3_ ceramics [121].

Unfortunately, the work [110] did not study spatial distribution of titania to reveal exact mechanisms of its influence. The grain size of TiO_2_ is too high (few microns) for 2DEG formed on heterointerface to contribute much to thermoelectric properties. Absence of data on TiO_2_ phase localization does not allow any conclusion on possible formation of an ordered 2DEG network in the composite material. Main difference in *ZT* values is caused by drastically different conductivity of the obtained materials. Possible reasons to this suggested by the authors in [110] are: Better conditions for ceramic grain growth as a result of surface modification with titania; possible contribution of the formed titanium carbide phase that is a better conductor than the oxides in the studied system. Also, there may be a contribution from 2DEG, which is likely formed on the heterointerface. In general, thermoelectric characteristics of TiO_2_-modified ceramics based on Sr(Ti_0.85_Nb_0.15_)O_3_ are close to the dense one Sr(Ti_0.8_Nb_0.2_)O_3_ [121]. TiO_2_ in that case plays a role of sintering additive. Abnormally low conductivity of the starting porous Sr(Ti_0.85_Nb_0.15_)O_3_ ceramics remains unclear, because it stays low in comparison with dense ceramics from [121] even after accounting for porosity in terms of dense ceramics. Summarizing the results of work [110], Sr(Ti_0.85_Nb_0.15_)O_3_-based composite ceramics modified with TiO_2_ is way too different in terms of structure compared to the biphase ceramics system we are suggesting (Figure 7). At least, the work [110] clearly demonstrates a positive influence of heterointerfaces (Figure 9) on thermal conductivity with the latter being considerably reduced.

Finally, one should pay attention to possible phase transition of titania (anatase–rutile) during sintering. 2DEG formation on TiO_2_/SrTiO_3_ heterointerface was considered here for the TiO_2_ anatase phase. However, formation of highly dense TiO_2_−SrTiO_3_ biphase ceramics requires sintering temperatures above 1000 °C. In 1967, A. Navrotsky and O. J. Kleppa observed phase transition anatase-rutile at 1050 °C when heating commercial anatase for 16 h [122]. Complete transition to rutile from anatase was demonstrated for ceramics sintering at 1100–1350 °C for 6 h [123] and at 1500 °C for 10 h [124]. Although here we suggest obtaining TiO_2_−SrTiO_3_ ceramics using spark plasma sintering, which implies by an order of magnitude shorter sintering durations, nevertheless, rutile formation is inevitable. In the next section, we will show that despite the conduction band edge of rutile being higher than that of anatase, strontium titanate is characterized by a higher conduction band edge anyway, which is the main factor for 2DEG formation on the heterointerface. Thus, possible rutile formation does not change the synthesis scheme of TiO_2_−SrTiO_3_ biphase ceramics, which intergranular boundary provides an ordered 2DEG network.

### 5.2. Quantum Chemical Analysis of TiO_2_/SrTiO_3_ Heterointerface Structure

**Figure 10:**
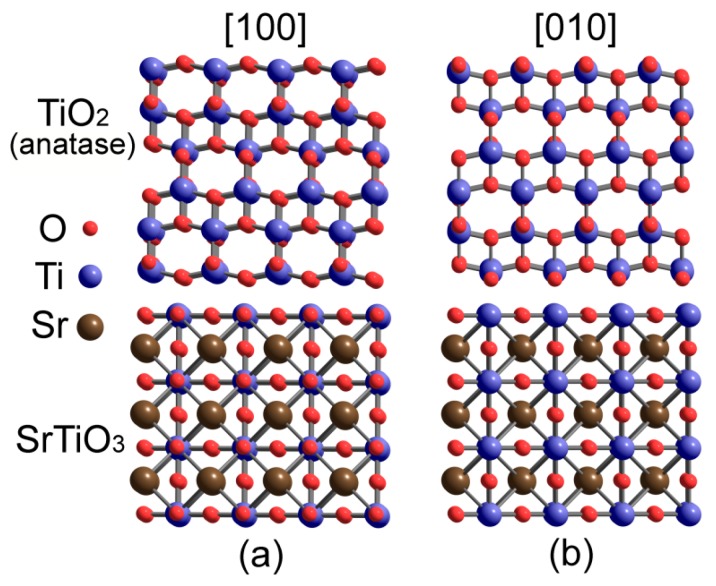
Structure of the heterointerface anatase TiO_2_ (001) on SrTiO_3_ (001) according to TEM image [125,126]. The views are along (**a**) the [100] and (b) the [010] directions.

According to TEM of epitaxial films of pure anatase TiO_2_ on undoped and *n*-type SrTiO_3_ [125,126], the most stable from an electrostatic standpoint is the structure of interface boundary given on Figure 10. However, this data does not provide enough information on heterointerface structure, e.g., about the edge of SrTiO_3_ support and how it is integrated with TiO_2_ film. That is why quantum chemical calculations is an important tool for studying electron structure of the heterointerface and how it correlates with thermoelectric properties.

Electronic structure of solids is frequently simulated via methods of density functional theory (DFT) [127,128], which are commonly applied with Khon–Sham formalism [129] using local density or the generalized gradient approximations (LDA and GGA, respectively) for the exchange-correlation functional. Modern DFT calculations often involve the projector augmented-wave (PAW) method [130,131], which combines simplicity of first-principles pseudopotential [132] and accuracy of linearized augmented planewave method [133]. PAW is an all-electron technique, which enables one to determine potential using all-electron density. That is why PAW proved to be as one of the most robust approximations for electronic structure modelling of crystals. Additionally, electronic structure of metal oxides is successfully simulated by the DFT+U technique, which was originally suggested [134] as a continuation of LDA within the Hubbard model [135]. As compared to hybrid functionals and resource-intensive post-Harthree–Fock methods (configurational interaction, Moller–Plesset perturbation theory, GW approximation and etc.), the DFT(LDA/GGA)+U approach provides equally correct results at fairly low computational cost. When optimal value of U = U_o_ − J are chosen (where U_0_ and J specifies the Coulomb and exchange interaction parameters, respectively [136]), one can obtain correct data on the electronic structure of solids as well as assess atomization potentials and energy of intermolecular interactions [137]. Simulations combined with the U correction can well describe the physical properties of periodic systems like magnetic and structural properties of correlated systems, the electron transfer energetics, and chemical reactions. The relative success of the DFT+U method owes its direct approach to accounting for the underestimated electronic interactions by the simple addition of a semiempirically tuned numeric parameter “U” [138] that can be easily controlled. This makes the DFT+U method a convenient tool to qualitatively assess the influence of electronic effects on physical properties of perovskites [139].

To reduce self-interaction error, in comparison to LDA and GGA, A.D. Becke suggested including a part of Hartree–Fock exchange energy into the в exchange functional [140] that had led to a great number of hybrid potentials, e.g., B3LYP [141] and PBE0 [142]. After implementation of plane wave codes [143] it became possible to use hybrid functionals for simulations of solid’s electronic structure that provided more accurate results (as compared to GGA) on lattice constants, atomization potentials, tensile energies and etc. However, to assess the Hartree–Fock exchange under periodic boundary conditions using plane waves one needs twice as many computational resources as compared to GGA.

**Figure 11:**
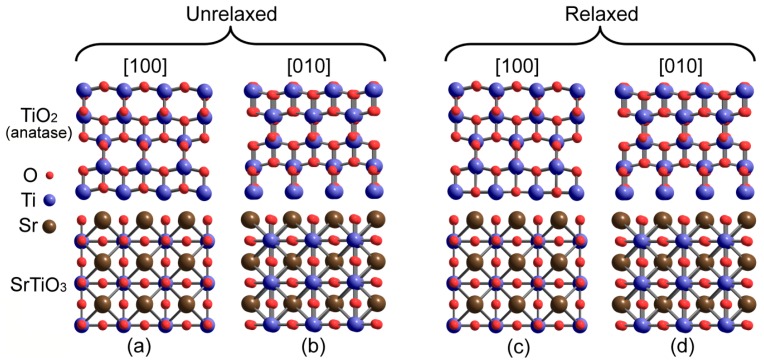
Structures of (**a**,**b**) unrelaxed and (**c**,**d**) relaxed interfaces between the SrO-terminated SrTiO_3_ and the Ti-terminated TiO_2_ according to [145]. Interface titanium atoms of TiO_2_ are located above hollow sites of SrTiO_3_ surface. The views are along (**a**,**c**) the [100] and (**b**,**d**) the [010] directions.

DFT/PAW modelling using the GGA PW91 functional [144] was used to study the atomic structure of the interface between SrTiO_3_ substrate and anatase TiO_2_ thin films [145] with results being in agreement with transmission electron and high-angle annular-dark-field microscopy data. Out of 24 possible interfacial geometries the most stable heterointerface structures were chosen, which were analyzed in terms of electron density distribution. Calculations by Z. Wang et al. have shown that the interface boundary contains SrO-terminated SrTiO_3_ and Ti-terminated TiO_2_, while the interfacial Ti atom from TiO_2_ is located above the hollow site (Figure 11) that is found to be the most energetically preferred. Besides, the modelling predicted mixed ion-covalent bonds in the TiO_2_/SrTiO_3_ interface as well as notable rearrangement of oxygen sublattice in the heterointerface. Namely, interfacial oxygen atoms of TiO_2_ are pulled towards the SrO plane of the SrTiO_3_, flattening the original zigzag of the TiO_2_ atomic chain.

**Figure 12:**
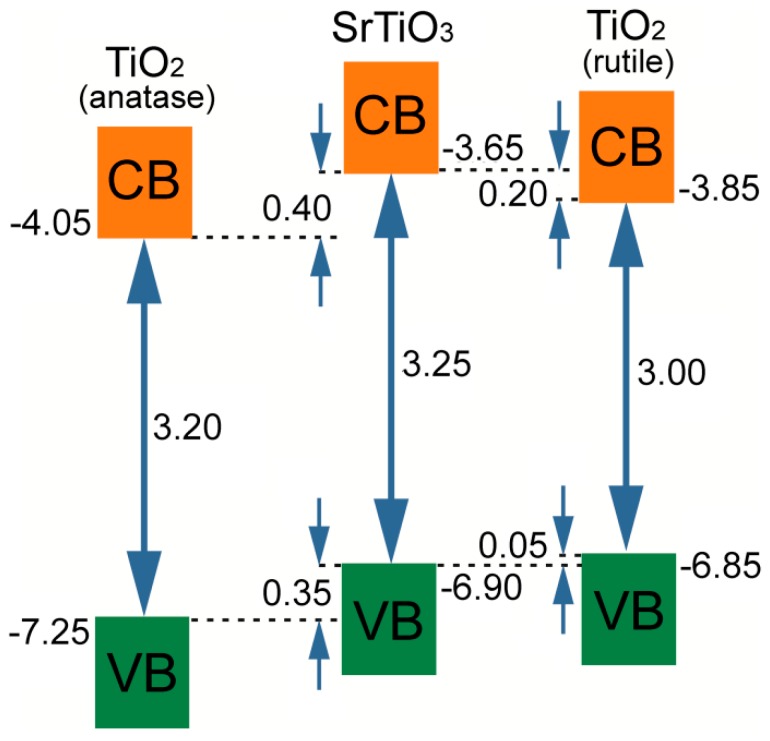
Energy level diagram (eV) of anatase, rutile, and SrTiO_3_ nanoparticles according to [98,147]. CB and VB stand for conduction band and valence band, respectively.

Theoretical investigations of the TiO_2_/SrTiO_3_ heterointerface presented above are carried out for anatase modification of titania. However, TiO_2_ grain structure in the biphase TiO_2_–SrTiO_3_ ceramics may correspond to rutile modification (See Section 5.1). That is why it is necessary to consider 2DEG formation on the TiO_2_(rutile)/SrTiO_3_ interfacial boundary. In accordance with the results of DFT/PAW simulations using three GGA exchange-correlation functionals for three potential epitaxial orientations between TiO_2_ and SrTiO_3_, the bond is observed only for the interface rutile TiO_2_ (001) on SrTiO_3_ (111) [146]. Experimental results from [98,147] show that conduction band edge of SrTiO_3_ is higher by 0.40 and 0.20 eV than that of anatase and rutile, respectively (Figure 12).

Namely, there is an opportunity to obtain 2DEG (modulation doping) on the boundary between thin films of rutile TiO_2_ (001) and SrTiO_3_ (111) that is also true for interface with anatase TiO_2_ (see Section 4.1). At the same time, modelling atomic and electronic structure of the interface rutile TiO_2_ (001) on SrTiO_3_ (111), according to [145], will enable us to obtain important results on structure of SrTiO_3_/TiO_2_ (rutile) heterointerface with various alloying agent to SrTiO_3_ and/or TiO_2_.

Regarding the preparation of fine-grained thermoelectric ceramics, one may also need calculations of the molecular structure of interacting nanoparticles to interpret experimental data. For example, the paper [148] reported the investigation of molecular structure of spherical TiO_2_ nanoparticles of various sizes (from 300 to 1000 atoms) using self-consistent charge density functional tight-binding (SCC-DFTB) and DFT (B3LYP). From an electronic structure standpoint, the accuracy of the bandgap energy calculations was assessed (Table 1). SCC-DFTB approximation was shown to provide rather accurate geometry and electronic structure of real sized TiO_2_ nanospheres (up to 4.4 nm).

Being in good agreement with the results of GW approximation, SCC-DFTB was successfully used to investigate the effects of ferroelectric domain walls on electronic transport properties and charge carrier recombination in organometallic halide perovskites [155]. Besides, the SCC-DFTB method was demonstrated to be reliable for a variety of TiO_2_ modifications [156,157]. Namely, SCC-DFTB modelling molecular structure of interacting nanoparticles of TiO_2_ and SrTiO_3_ as well as simulating their grain structure with alloying agent will enable to predict their optimal size and type/quantity of the agent, respectively. This is an indispensable step towards targeted engineering of TiO_2_–SrTiO_3_ ceramic thermoelectrics with maximal *ZT* value.

### 5.3. TiO_2_–SrTiO_3_ Biphase Nanoceramics: SPS Approach

As was mentioned earlier, consolidation (sintering) methods gain specific importance when it comes to fabrication of nanostructured thermoelectrics. On the one hand, it is necessary to obtain highly-dense material, on the other hand, preserving small (nanoscale) grain size is crucial. Such material’s microstructure is important to improve its electrical conductivity and reduce thermal conductivity that is desired for thermoelectric applications. A variety of sintering techniques have been used in the literature to achieve SrTiO_3_-based bulk samples. However, hot pressing (HP) and spark plasma sintering (SPS) are the most commonly used techniques for making high-density (typically >95% of the theoretical density) and small crystal size (<1 µm) SrTiO_3_-based bulk samples (Table 2). Accordingly to Table 2, the maximum dimensionless figure of merit obtained by A. Kikuchi et al. in SPSed 8 at.% La-doped SrTiO_3_ (*ZT* = 0.37 at 1045 K) [158], and by Y. Wang et al. in HPed 5 at.% Gd-doped SrO(SrTiO_3_)_2_ (*ZT* = 0.24 at 1000 K) [159].

The concept of SPS (rapid heating under pressure with pulses of direct current) consists in rapid heating of nanopowders to sintering temperature at a high speed (to 250 °C/min) by short (order of ms) electric pulses under moderate pressure (<100 MPa). Such conditions favor grain-boundary and surface diffusion, i.e., densification dominates over grain growth. Short sintering cycle provides ceramics with submicron- and nano-grained structure with a density close to theoretical. Owing to high pulse frequency, total amount of energy transferred by spark discharges is comparable with energy transferred in the graphite die that provides uniform heating of the material yielding a uniform composition of the obtained product [160].

This effect is the main difference from the hot-pressing technology, which conducts the Joule heat to the material from the outside (from inductive or resistive heater). That results in a temperature gradient (temperature spatial non-uniformity) between the center and the periphery (surface) of ceramics, particularly at large sizes of green bodies, that may yield in non-uniform material in terms of chemical (phase) composition and microstructure. Additionally, hot pressing cycle takes a long time, because pressure can be applied only after holding for some time, which is necessary for temperature balancing along the sample. That is why we give the priority to the SPS method here.

Recently, D.Yu. Kosyanov et al. suggested a modified approach to fabrication of highly-dense fine-grained ceramics based on reactive SPS of nanopowders in the oxide systems with controlled particle size distribution, using Nd^3+^:YAG as an example [165]. This route allows implementing external pressure, particle surface curvature (nanopowders of different sizes) and chemical reactions (phase transformations according to the scheme “starting oxides → intermediate phases → final product”) simultaneously as driving forces of sintering to obtain uniform highly dense microstructure. Crystal lattice rearrangement during phase transitions activates diffusion-dislocation processes promoting effective densification of ceramics due to grain rotation and migration as a whole [166,167]. This allows the reduction of sintering time by 10–20 times as compared to non-reactive SPS of similar materials.

Given the abovementioned, the formation of biphase composite fine-grained TiO_2_–SrTiO_3_ ceramics should to be carried out via reactive SPS of strontium oxide SrO (formed by the decomposition of SrCO_3_) and titania TiO_2_. Checkered structure of ceramics will be provided by TiO_2_ and SrTiO_3_ phases taken in the ratio 50:50 vol.% that corresponds to the molar ratio of 73.835 mol.%:26.165 mol.%. Uniform distribution of the two phases will provide long-range mass transport between interface boundaries, which has a limiting effect of the atomic movement (excessive grain growth) [161]. Fabrication of highly-dense biphase ceramics with uniform distribution of two phases was demonstrated for Al_2_O_3_–Y_3_Al_5_O_12_, Y_2_O_3_–MgO system [168,169,170].

SrTiO_3_ formation proceeds in at least three stages, which was reported in detail here [171]. Phase and closed-type porosity formation is preceded by the sample’s heating, which embraces the decomposition of strontium carbonate SrCO_3_ to strontium oxide SrO. Mass transfer in the depth of the product for the SrO–TiO_2_ system is limited by titanium cations, which is in agreement with Sr–O and Ti–O bond strengths in SrTiO_3_: Ti^4+^ ions are two times smaller than Sr^2+^ ones (*r*(Ti^4+^) = 0.605 Å (6); *r*(Sr^2+^) = 1.26 Å (8)).

Because reaction rate constant is inversely proportional to the radius of particles coated with agent (TiO_2_ in our case) during sintering, particle size of the starting powder will play a major role in the kinetics of SrTiO_3_ phase formation and densification. Niwa et al. reported a high-temperature gravimetric study on the kinetics of formation of SrTiO_3_ [172]. The reactivity of starting materials and the kinetics of the reaction of strontium carbonate with titanium dioxide have been studied. The reaction of strontium carbonate SrCO_3_ with rutile TiO_2_ obeyed the nuclei growth rate equation, with an activation energy of 409 kJ∙mol^−1^. The kinetics of the reaction between strontium carbonate SrCO_3_ and anatase TiO_2_ was described by a diffusion-controlled rate equation with a much lower activation energy of 279 kJ∙mol^−1^. The difference between rutile and anatase in reacting with strontium carbonate has been related to the closer unit cell parameters of anatase and SrTiO_3_ [173]. Thus, it is anatase TiO_2_ nanopowders that are important to use for increasing the point of contact between starting materials and decreasing the activation energy to improve the reaction rate.

**Figure 13:**
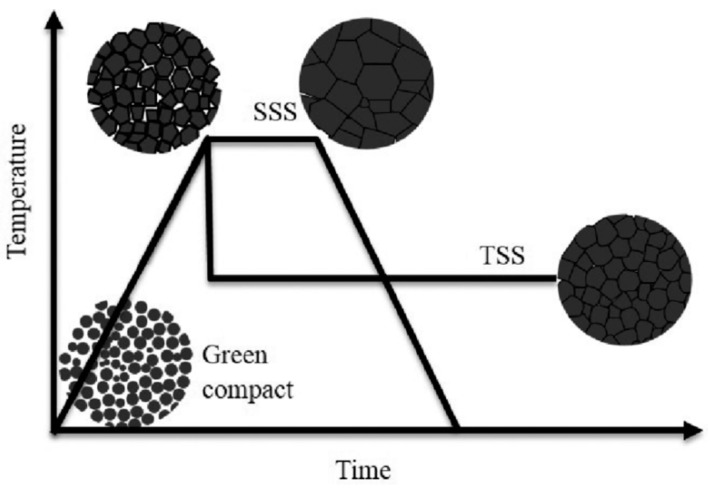
Schematic of densification of ceramics with grain growth during conventional single-step sintering (SSS) and densification without grain growth at lower second-step temperature during TSS with higher first-step temperature. © 2017 Sutharsini U, Thanihaichelvan M, Singh R. Published in Ref. [175] under CC BY 3.0 license. Available from: https://doi.org/10.5772/68083.

It is suggested that two-stage sintering (TSS) within reactive SPS to suppress the grain growth should be implemented. For example, in [174], non-reactive SPS was performed stepwise, i.e., the sample was primarily heated to 1220 °C and then immediately cooled down to 1000 °C followed by dwelling for 15 min without external pressure. As compared to ordinary “single-step” SPS (SSS) at 1250 °C for 30 min, authors obtained ceramic with nearly the same density of ~95% from theoretical. But for SrTiO_3_, grains in samples sintered by TSS were some 40% smaller than in the samples sintered by the SSS method (0.35 µm vs. 0.56 µm). This effect is clearly illustrated by the scheme in Figure 13 [175]. The authors claimed in [174] that even larger differences in grain sizes obtained by TSS and SSS might be expected at higher final densities than 95%TD, when a uniaxial pressure is additionally used.

It is noteworthy that sintering in vacuum will initiate the formation of oxygen vacancies *V*_O_′′ due to extraction of O^2−^ ions from TiO_2_ and SrTiO_3_ structures. At the same time, concentrating limited electron-carriers occur most likely on TiO_2_/SrTiO_3_ interfaces as in the most loose, defect regions of TiO_2_ and SrTiO_3_ crystallites. On the other hand, the results of [176] indicate that intrinsic point defects in the pure SrTiO_3_ ceramics are mainly *V*_Ti_′′′′. Probably, V_O_′′ will be associated with a cation vacancy forming (V_Ti_′′′′ + 2V_O_′′) complex, namely, the Shottky defect. Federicci et al. in [177] reported a superionic conductor with a colossal dielectric constant for Rb_2_Ti_2_O_5_ materials annealed under an oxygen-depleted atmosphere (such as He, N_2_, vacuum). They found that oxygen vacancies are created in the material, and the dielectric constant reaches the unprecedented value of 10^9^. They proposed a possible mechanism of a Frenkel anionic defect. Additionally, they pointed out that another reason for such a high dielectric constant is that at least two ionic species of opposite signs are involved in the process. It was reported in the previous sections that oxygen vacancies are equivalent to n-doping to some extent [99], which is very important for 2DEG formation of heterointerfaces [27]. In other words, the formation of point defects in the TiO_2_/SrTiO_3_ structure will serve as additional factor enhancing the *ZT* value.

## 6. Summary and Future Prospect

Analysis of theoretical and experimental works has shown that multiple boundaries improves thermoelectric properties of the material because of two reasons, namely: Phonon filtration to drag only electrons (important for low temperatures) or simply intense phonon dissipation; 2DEG formation on the boundaries that filters electrons with respect to energy and possesses high thermoelectric characteristics itself. Structures able to generate 2DEG are produced by the only means of laser epitaxy that are hardly suitable for large scale manufacturing. Theoretical modelling of electronic structure suggests considering honeycomb architecture, which utilizes 2DEG features, yielding a drastic increase of the material’s thermoelectric properties. But such architecture implies the special formation of thin films of the phase localizing 2DEG, which is a challenging and unresolved task. However, it is not necessary to build a 2D phase to localize 2DEG, because it can be formed on the interface between two oxide phases. This fact was not paid attention to in previous works related to materials with 2DEG.

That is why this review suggests to implement biphase ceramics based on SrTiO_3_ and TiO_2_. Its main feature is that 2DEG occurs spontaneously on the TiO_2_/SrTiO_3_ heterointerface, unlike ordinary 2DEG formation on specially formed thin film, because the conduction band edge of SrTiO_3_ is 0.40 and 0.20 eV higher than that for anatase and rutile TiO_2_, respectively. According to quantum chemical modelling of TiO_2_(anatase)/SrTiO_3_ and TiO_2_(rutile)/SrTiO_3_, chemical bonds were shown to appear on the interface for both crystal modifications. That is why the checkered order of TiO_2_ and SrTiO_3_ grains in the material’s bulk will probably provide the single connected 2D surface along grain boundaries with 2DEG. Additionally, application of the SCC-DFTB method for modelling molecular structure of TiO_2_ and SrTiO_3_ interacting nanoparticles will enable us to predict optimal grain size and type/quantity of alloying agents for thermoelectrics with maximal *ZT* value.

The promising method of reactive SPS of anatase TiO_2_ and SrO (SrCO_3_) nanopowders is suggested for the preparation of highly dense material preserving the small (nanoscale) grain size of TiO_2_, SrTiO_3_ and thin interface layer between them. Uniform distribution of the two phases will provide long-range mass transport between interface boundaries, which has a limiting effect of the atomic movement (excessive grain growth). Fabrication of TiO_2_−SrTiO_3_ biphase nanoceramics will demonstrate that rapid sintering under pressure of two-phase oxide ceramics is the key towards the development of thermoelectric materials, utilizing 2DEG properties, for high-temperature applications.

## Figures and Tables

**Table 1:** Electronic band gap for bulk anatase TiO_2_. Self-consistent charge density functional tight-binding (SCC-DFTB) values have been compared to the experimental data, values calculated with standard GGA-DFT, with hybrid DFT functionals, GGA-DFT Hubbard corrected and post-Hartree–Fock GW approximation [149].

Method	PBE	PBE0	B3LYP	PBE+U	GW	DFTB	Experiment
Band gap, eV	2.36 [150]	4.50 [151]	3.81 [152]	3.27 [151]	3.83 [153]	3.22 [148]	3.4 [154]

**Table 2:** Lists the hot pressing (HP) and spark plasma sintering (SPS) parameters employed to prepare SrTiO_3_-based ceramics reported in the literature by several groups.

	Authors	Composition	Sintering Conditions	Pressure	Density	Largest Figure of Merit	Ref.
1	H. Obara et al.	10 at.% Y-doped SrTiO_3_	HP @ 1400 °C, 1 h	100 MPa (Argon)	>97%	*ZT* = 0.10 at 490 K	[159]
2	M. Ito and T. Matsuda	SrTiO_3_	HP @ 1400 °C, 2 h	25 MPa (Argon)	95.8%	*ZT*~0.092 at 870 K	[161]
		10 at.% Y-doped SrTiO_3_			97.2%	*ZT* = 0.146 at 870 K	
3	Y. Wang et al.	5 at.% Gd-doped SrO(SrTiO_3_)_2_	HP @ 1450 °C, 1 h	36 MPa (Argon)	96–99%	*ZT*~0.24 at 1000 K	[162]
		5 at.% Nb-doped SrO(SrTiO_3_)_2_				*ZT* = 0.14 at 1000 K	
4	A. Kikuchi et al.	8 at.% La-doped SrTiO_3_	SPS @ 1300 °C, 5 min	34 MPa (Vacuum)	97.7%	*ZT* = 0.37 at 1045 K	[158]
5	P.-P. Shang et al.	8 at.% La-doped SrTiO_3_	SPS @ 1200 °C, 5 min	40 MPa (Vacuum)	91.6%	*ZT* = 0.08 at 679 K	[163]
6	N. Okinaka et al.	8 at.% La-doped SrTiO_3_	SPS @ 1300 °C, 30 min	34 MPa (Vacuum)	>95%	*ZT* = 0.22 at 800 K	[164]
